# Surgical management and adverse factors for recurrence and long-term survival in adenoid cystic carcinoma patients with intracranial extension: A retrospective cohort study

**DOI:** 10.1097/MD.0000000000043888

**Published:** 2025-08-15

**Authors:** Xu-Lei Huo, Da Li, Xi-Chao Yin, Zhen Wu, Ke Wang

**Affiliations:** a Department of Neurosurgery, Beijing Tiantan Hospital, Capital Medical University, Beijing, China.

**Keywords:** chemotherapy, clinical characteristics, intracranial adenoid cystic carcinomas, radiotherapy, skull base

## Abstract

Limited data regarding intracranial adenoid cystic carcinomas (ACCs) were available. The authors aimed to elaborate on the clinical characteristics, treatment strategy, and poor outcomes of the disease. Clinical data from all cases of intracranial ACCs treated at our institute were reviewed retrospectively to evaluate their clinical characteristics, management, and outcomes. our series included 10 males and 9 females, with a mean age of 47.3 years. The most common presentations were cranial neuropathies (11 cases), followed by headache (4 cases), and nasal symptoms (4 cases). The radiologic spectrum for intracranial ACCs was broad. Gross total resection (GTR), subtotal resection, and partial resection were performed in 8 (42.1%), 6 (31.6%) and 5 (26.3%) patients, respectively. After a median follow-up of 35.0 months (range, 3.0–89.0 months), 8 patients (42.1%) died. The 1-, 3-, and 5-year rates of progression-free survival and overall survival were 89.2%, 57.3%, and 20.5% and 89.5%, 57.5%, and 32.9%, respectively. Although the differences were not significantly different, GTR and the use of radiotherapy and chemotherapy tended to improve the prognosis of the patients. Intracranial ACCs are rare neoplasms. GTR alone, if tolerable, is advocated as the optimal treatment for intracranial ACCs. Nevertheless, conservative excision may be preferred to avoid damage to vital structures. Radiotherapy and chemotherapy may be an alternative treatment. Intracranial ACCs tend to recur or metastasis within a few years of the initial surgery.

## 1. Introduction

Adenoid cystic carcinomas (ACCs), which were first described as a “cylindroma” by Billroth, are rare, histologically malignant tumors derived from secretory epithelium tissues of the minor and major salivary glands, nasopharynx, lacrimal gland, lung, trachea, mammary gland, and skin.^[1,2]^ It constitutes 8 to 10% of all salivary gland tumors, and the frequency of intracranial spread ranges from 4% to 22%.^[3,4]^ Limited data regarding intracranial ACCs are available in the literature, and most cases have been presented as isolated and anecdotal case reports. Given the paucity of information regarding these tumors in the published literature, their management and clinical characteristics remain ambiguous. No researcher has yet investigated this variant exclusively to better understand the clinical characteristics, optimal management, and outcomes of this disease. Therefore, we retrospectively reviewed data on all patients who had undergone surgical treatment at our institute for pathologically confirmed ACC.

Here, we reported the largest series to date of 19 cases of intracranial ACCs located in the skull. In this study, we will describe the clinical features of intracranial ACCs with a review of the literature. Besides, progression-free survival (PFS) and overall survival (OS) were assessed to ascertain the efficacy of surgery, radiotherapy (RT), and chemotherapy (CMT). Lastly, a discussion of optimal management is based on our findings and other reports.

## 2. Methods

### 2.1. Study population and data collection

This retrospective study included consecutive patients with histologically confirmed ACC treated at our institution between January 2010 and December 2023. The study protocol received approval from the Beijing Tiantan Hospital Research Ethics Committee. Inclusion criteria: Histopathological confirmation of ACC; tumor location restricted to intracranial and/or skull base regions; availability of complete treatment records and follow-up data including: therapeutic interventions, neurological outcomes, recurrence status, and survival data. Exclusion criteria: Extracranial primary tumors; incomplete clinical documentation; nonclinical studies (e.g., basic science research); cases with ambiguous pathological diagnosis.

A comprehensive review of medical records was conducted, including: demographic characteristics (age, sex), clinical presentation (primary symptoms, duration), preoperative functional status (Karnofsky Performance Score), surgical parameters (extent of resection) and tumor characteristics, which including anatomic location, radiographic features (MRI/CT), and volumetric assessment using orthogonal diameters. And the tumor size calculated as (*a* × *b* × *c*)^(1/3), where *a* = maximal axial diameter, *b* = maximal coronal diameter, and *c* = maximal sagittal diameter.

Surgical intervention aimed to achieve optimal tumor debulking while maintaining the integrity of surrounding neural and vascular tissues. The operative strategy was customized according to each patient’s specific anatomical and pathological characteristics. The extent of surgery (ETR) was classified according to the evaluations (cubature formula: volume = [*a* × *b* × *c*]/2) of both pre- and postoperative tumor volumes based on contrast MRI scans, as follows: gross total resection (GTR) for no residue was observed on postoperative MRI; subtotal resection (STR) for 90% to 99% tumor removal, and partial resection (PR) for cases with < 90% resection volume.

Postoperative surveillance included scheduled clinical evaluations and radiographic assessments at 3-month intervals during the initial 2-year period, followed by biannual monitoring for patients without evidence of disease progression. Cases demonstrating tumor recurrence or progression were maintained on a 3-month follow-up regimen. All patients received postoperative RT or CMT, with treatment adherence and neurological function systematically documented during follow-up visits. Radiographic progression was quantitatively defined as a ≥ 2 mm increase in maximal tumor diameter on serial MRI examinations. Survival outcomes were calculated from the date of definitive surgical intervention, with PFS representing the interval to either radiologically confirmed recurrence or death, and OS reflecting time to death or last clinical contact.

## 3. Literature search

We conducted a systematic literature search using the MeSH term “adenoid cystic carcinoma” across multiple databases (OVID, MEDLINE, EMBASE, PubMed, and Cochrane Database) for publications from 1996 to 2018. The search was supplemented by manual review of references from eligible articles to identify additional cases. Inclusion/exclusion criteria: Included: Cases with primary intracranial ACC; excluded: Cases with synchronous malignancies (e.g., glioma); extracranial rhabdoid tumors (e.g., renal lesions); duplicate case reports. Two independent investigators (KW and DL) performed the screening process, verifying the source and publication date of each case to eliminate redundancies. Discrepancies were resolved through consensus discussion.

## 4. Immunohistochemical examination

Following surgery, tumor specimens were fixed in paraffin and sectioned at 5 μm thickness for hematoxylin and eosin (H&E) staining. Immunohistochemical analysis was performed with relevant markers, including cytokeratin, epithelial membrane antigen (EMA), glial fibrillary acidic protein (GFAP), S-100, vimentin, Ki-67, CEA, cytokeratin 8/18, cytokeratin 5/6, smooth muscle actin (SMA), and calponin to facilitate differential diagnosis. All histopathological assessments were independently reviewed by a senior neuropathologist (JD). Final diagnoses were confirmed based on characteristic morphological features consistent with ACC, including its distinctive cribriform architecture and basaloid cell patterns.

## 5. Statistical analysis

The primary outcome for patients with intracranial ACCs was PFS and OS. The pertinent risk factors were evaluated with univariate and multivariate Cox regression analysis of the cases (total 19 cases). Kaplan–Meier analysis was used to assess OS and PFS after surgery; a log-rank test was used to assess the effect of ETR, RT, or CMT on OS or PFS. All statistical analyses were performed in R version 4.0.5, utilizing the survival package (Terry M. Therneau, Patricia M. Grambsch) for survival modeling, survminer (Alboukadel Kassambara and Marcin Kosinski) for survival curve visualization, and ggplot2 (Hadley Wickham) for graphical representations. A *P*-value threshold of .05 was defined statistical significance.

## 6. Results

### 6.1. Illustrative cases

#### 6.1.1. Case 1

**Figure 1.**
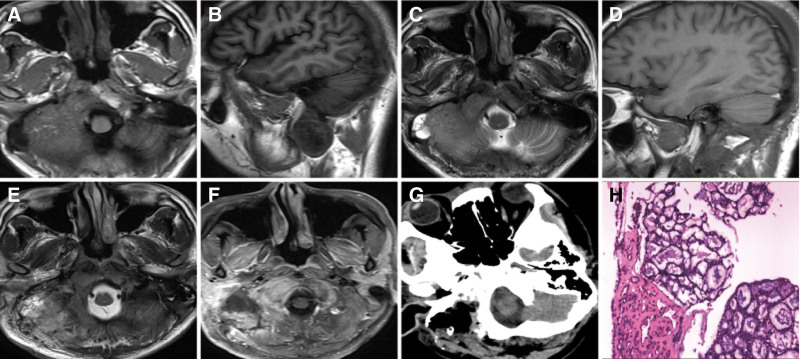
Case 1. Magnetic resonance images showing isointense T2 (A) and isointense T1 (B and C) signals on the right jugular foramen. Magnetic resonance images (D–F) and axial CT (G) revealing gross total resection. Pathological examination (H) revealed the diagnosis of ACC. H & E, original magnification = 200×. ACC = adenoid cystic carcinoma, CT = computed tomography.

An irregular lesion occupying the right jugular foramen and neck was radiologically diagnosed in a 28-year-old man admitted to our hospital. He reported a stiff neck for 6 months, difficulty swallowing and drinking for 40 days, and shoulder pain for the past month. Neurological examination revealed the peripheral facial paralysis on the right side, tongue extended to the right, and difficulty neck rotation and shrug. Radiological examination revealed a lesion with isointense T1 and isointense T2 signals (Fig. 1). The patient underwent a right far lateral approach to gross remove the tumor. During the surgery, the tumor appeared as a grayish-white tenacious mass with a poorly defined border. Postoperative pathology revealed a diagnosis of ACC (Fig. 1). The patient was discharged 14 days after surgery without any neurological deterioration.

#### 6.1.2. Case 2

**Figure 2.**
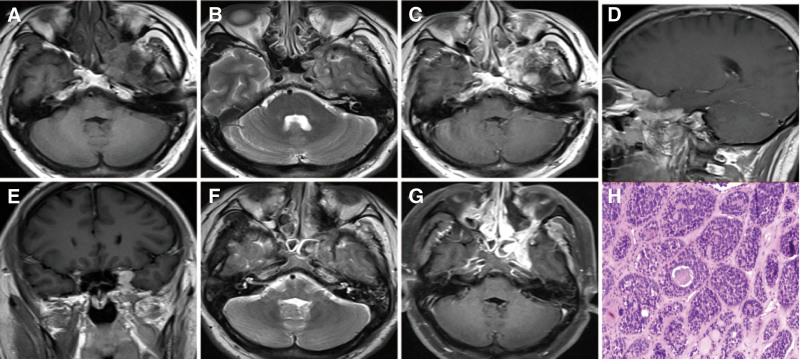
Case 2. Magnetic resonance images showing isointense T2 (A), isointense T1 (B) in the left para-saddle, sphenoid condyle, maxillary sinus, and eye socket with the homogeneous enhance (C–E). Magnetic resonance images (F and G) revealing partially remove the tumor. The staining of ACC (H) revealed the round or oval tumor cells, similar to basal cells, and aggregated in pellets. H & E, original magnification = 200×. ACC = adenoid cystic carcinoma.

A large flat lesion occupying the left para-saddle, sphenoid ridge, maxillary sinus, and orbit was radiologically diagnosed in a 36-year-old man admitted to our hospital. He reported a progressive increase in the sensory disturbances in the left maxillofacial region for the past 4 years. Neurological examination revealed a loss of vision and abduction in the left eye and diplopia. Radiological examination revealed a lesion with isointense T1 and isointense T2 signals as well as homogeneous enhance (Fig. 2). This lesion showed hyper-density on computed tomography (CT) examination with the bone destruction. The patient underwent an endoscopic trans-nasal approach to partially remove the tumor. During the surgery, the tumor appeared as a grayish-yellow tenacious mass with a poorly defined border. Postoperative pathology revealed a diagnosis of ACC (Fig. 2). Immunohistochemical staining revealed that cytokeratin, cytokeratin 8/18, EMA, calponin, SMA, and S-100 were positive. The patient was discharged 8 days after surgery without any neurological deterioration.

#### 6.1.3. Case 3

**Figure 3.**
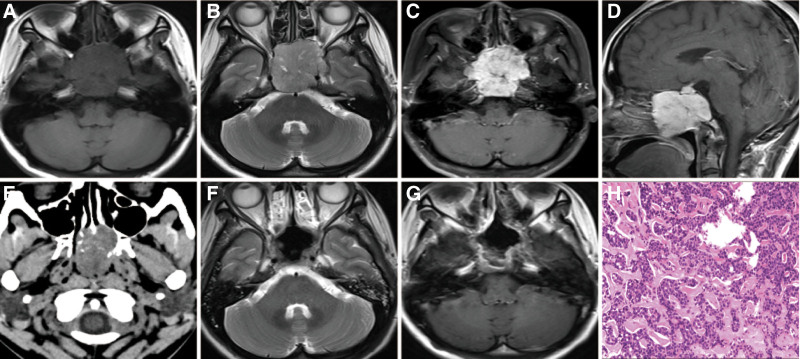
Case 11. Magnetic resonance images showing isointense T2 (A) isointense T1 (B) in the slope and sphenoid sinus and with the heterogeneous enhance (C and D). Bone destruction and the mixed-density lesion were observed on computed tomography (E). Magnetic resonance images (F and G) revealing gross remove the tumor. Pathological examination (H) revealed the diagnosis of ACC. H & E, original magnification = 200×. ACC = adenoid cystic carcinoma.

An irregular lesion occupying the clivus, and sphenoid sinus was radiologically diagnosed in a 37-year-old female admitted to our hospital. She reported alternating nasal congestion for 7 months and a headache for the past 6 months. Neurological examination revealed no neurological function loss. Radiological examination revealed a lesion with isointense T1 and isointense T2 signals (Fig. 3) as well as heterogeneous enhance. This lesion showed mixed-density on CT examination with the bone destruction (Fig. 3). The patient underwent an endoscopic trans-nasal approach to gross totally remove the tumor. During the surgery, the tumor appeared as a grayish-red tenacious mass with a well-defined border. Postoperative pathology revealed a diagnosis of ACC (Fig. 3). Immunohistochemical staining revealed that cytokeratin, cytokeratin 5/6, calponin, and S-100 were positive while the value of Ki-67 is above 10%. The patient was discharged 7 days after surgery without any neurological deterioration.

#### 6.1.4. Case 4

**Figure 4.**
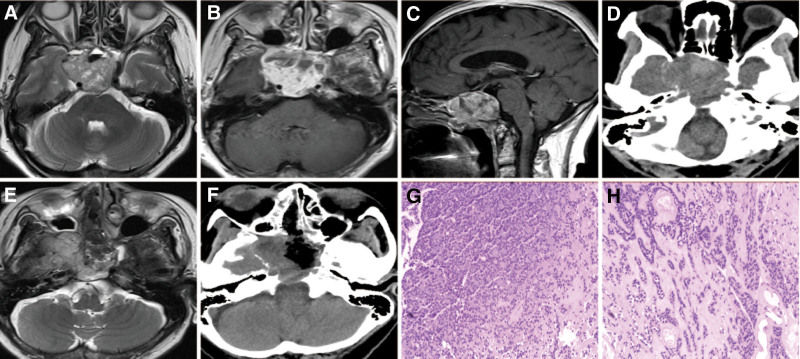
Case 12. Preoperative MRIs (A–C) showed a lesion located in the right wing-fossa, sphenoid bone, parapharyngeal, and mixed-signal and mixed-signal on T2-weighted imaging, and heterogeneous enhancement on contrast MRI. ACC can manifest as mixed-density with the bone destruction and the tumor calcification on preoperative CT scans (D). Postoperative MRI and CT scans showed resection partially (E and F). Staining of ACC (G and H) revealed the round or oval tumor cells, similar to basal cells, and aggregated in pellets or pellet-shaped mucus with one or more layers of tumor cells around it, H & E, original magnification = 100× and 200×. ACC = adenoid cystic carcinoma, CT = computed tomography, MRI = magnetic resonance image.

An irregular lesion occupying the right wing-fossa, sphenoid bone, parapharyngeal was radiologically diagnosed in a 56-year-old female admitted to our hospital. She reported progressive visual acuity decline in the right eye with frequent drinking and urination for the past 2 months. Neurological examination revealed that the right eye only had a light vision. Radiological examination revealed a lesion with mixed T1 and mixed T2 signals (Fig. 4) as well as heterogeneous enhance. This lesion showed mixed-density on CT examination with bone destruction and tumor calcification (Fig. 4). The patient underwent an endoscopic trans-nasal approach to partially remove the tumor. During the surgery, the tumor appeared as a grayish-red tenacious mass with a poorly defined border. Postoperative pathology revealed a diagnosis of ACC (Fig. 4). Immunohistochemical staining revealed that cytokeratin, cytokeratin 5/6, EMA, calponin, vimentin, and S-100 were positive while the value of Ki-67 was 1% to 5%. Furthermore, the CEA, CK8/18, PRL, PR, ER, and SYN were negative. The patient was discharged 13 days after surgery without any neurological deterioration.

#### 6.1.5. Case 5

**Figure 5.**
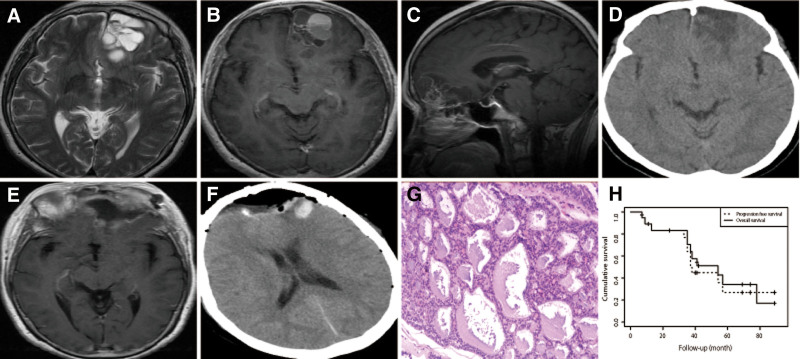
Case 19. Preoperative MRIs (A–C) showed a lesion located in the left frontal lobe and hypointense signal and hyperintense signal on T2-weighted imaging and heterogeneous enhancement on contrast MRI. ACC can manifest as mixed – density with the bone destruction on preoperative CT scans (D). Postoperative CT scans (E) and MRIs (F) showed gross total resection. Staining of ACC (G) revealed the round or oval tumor cells, similar to basal cells, and aggregated in pellets or pellet-shaped mucus with one or more layers of tumor cells around it, H & E, original magnification = 200×. Kaplan–Meier plot (H) illustrating progression-free survival and overall survival of the overall cohort (n = 19). ACC = adenoid cystic carcinoma, CT = computed tomography, MRI = magnetic resonance image.

An irregular lesion occupying the bilateral nasal cavity and left frontal lobe was radiologically diagnosed in a 58-year-old female admitted to our hospital. She reported intermittent bleeding on the right nasal for the past 4 years. She had nasal bleeding 4 years ago, and a biopsy showed that the ACC with postoperative treatment of gamma knife. Neurological examination revealed that the weak smell on the right side. Radiological examination revealed a lesion with hypointense T1 and hyperintense T2 signals (Fig. 5) as well as heterogeneous enhance. This lesion showed mixed-density on CT examination with the bone destruction (Fig. 5). The patient underwent a double frontal approach to gross totally remove the tumor. During the surgery, the tumor appeared as a grayish-red tenacious mass with a poorly defined border. Postoperative pathology revealed a diagnosis of ACC (Fig. 5). Immunohistochemical staining revealed that cytokeratin 5/6, EMA, CEA, calponin, GFAP, and S-100 were positive while the cytokeratin 8/18 and AFP were negative. The patient was discharged 13 days after surgery without any neurological deterioration.

## 7. Patient demographics

The clinical characteristics of all 19 patients are summarized in Tables 1 and 2. The cases included 10 males and 9 females, with a mean age of 47.3 years (range 24.0–63.0 years). The mean duration of symptoms was 17.7 months (range 2.0–60.0 months), and the most common preoperative symptoms were cranial deficits (12 cases), followed by headache (4 cases), and nasal symptoms (4 cases). The median preoperative KPS score was 81.6, the median postoperative KPS score was 74.2, and the follow-up KPS was 46.3. Cranial neuropathies were observed in 12 (63.2%) patients, and cranial nerve (CN) II palsy (7 cases) was the most common type, followed by CN V (5 cases), CN III (1 case), CN IV (1 case), CN VI (1 case) palsy, CN VIII (1 case), CNX (1 case), CNXI (1 case), CN XII (1 case), and CNXIII (1 case). Five patients had undergone surgical treatment before being admitted to our institute (Table 1).

**Table 1:** Patients demographics of adenoid cystic carcinoma cases.

No.	Sex/age (yr)	Signs and symptoms	Location	Treatment	PFS (mo)	OS (mo)
1*	58/F	CN II, III, and IV	Saddle area	S + RT	24	24†
2	28/M	CN X, XI, XII and XIII	Right Jugular foramen and neck	S	11	11†
3	38/F	Headache, CN V	Left para-saddle and orbital apex	S + RT + CMT	29	29
4	36/M	CN II, VI, and V	Left para-saddle, sphenoid ridge, (involving maxillary sinus and orbit)	S + RT	40	42†
5	53/F	CN II and V	Clivus, saddle, left sphenoid sinus, pterygopalatine fossa	S + RT	48	48
6	57/M	Headache and nasal bleeding	Left para-saddle (involving maxillary sinus)	S	7	7
7*	53/F	Postoperative ACC	Frontal lobe	S + RT	41	41†
8*	63/M	CN V and nasal bleeding	Right maxillary sinus, pterygopalatine fossa and para-saddle	S + RT + CMT	74	74†
9	51/M	Dizziness, nausea, and vomiting	Left temporal lobe	S	7	7†
10	57/M	CN V	Posterior pharyngeal wall	S + RT + CMT	31	31
11	37/F	Nasal congestion and headache	Clivus, sphenoid sinus	S	51	51
12	56/F	CN II, Polydipsia and polyuria	Right wing fossa, sphenoid bone, parapharyngeal	S	89	89†
13	24/F	Headache	Left middle cranial fossa and para-saddle	S	33‡	35
14	47/M	CN II	Left orbital apex, cavernous sinus	S + RT	37‡	72
15*	50/M	Nasal bleeding	Right nasal cavity	S	48	48
16	28/F	CN V	Left saddle, orbital apex, pterygopalatine fossa	S + RT + CMT	69	69†
17	62/M	Dizziness and CN II	Saddle area and left cavernous sinus	S + RT	3	3
18	42/M	CN II and VIII	Left infratemporal fossa, cavernous sinus, orbital apex	S + RT	29	29
19*	58/F	Nasal bleeding	Bilateral nasal cavity and left frontal lobe	S + RT	32	32

CN = cranial nerve, CMT = chemotherapy, F = female, M = male, OS = overall survival, PFS = progression-free survival, RT = radiotherapy, S = surgery.

* The patient had undergone surgical treatment before being admitted to our institute.

† Patient died finally.

‡ Tumor progression was observed during follow-up.

**Table 2:** Clinical characteristics of patients with adenoid cystic carcinoma.

Variables	n (%)
Age at diagnosis (yr)	
Median (range)	51.0 (24.0–63.0)
Mean ± SD	47.3 ± 12.2
Sex, no. (%)	
Male	10 (52.6)
Female	9 (47.4)
Size	
Median (range)	37.2 (16.4–56.2)
Mean ± SD	35.3 ± 12.2
Main complaint, no. (%)	
Cranial nerve deficits	12 (63.2)
CN II	7 (36.8)
CN V	5 (26.3)
Headache	4 (21.1)
Nasal symptoms	4 (21.1)
Duration of symptoms in mo	
Median (range)	12.0 (2.0–60.0)
Mean ± SD	17.7 ± 18.6
Postoperative radiotherapy, no. (%)	
Yes	12 (63.2)
No	7 (36.8)
Postoperative chemotherapy, no. (%)	
Yes	4 (21.1)
No	15 (78.9)
Resection, no. (%)	
GTR	8 (42.1)
STR	6 (31.6)
PR	5 (26.3)
Preoperative KPS	
Median (range)	90.0 (40.0–90.0)
Mean ± SD	81.6 (13.8)
Postoperative KPS	
Median (range)	80.0 (20.0–90.0)
Mean ± SD	74.2 (21.2)
FU KPS	
Median (range)	80.0 (0.0–100)
Mean ± SD	46.3 (45.4)
FU duration (mos)	
Median (range)	35.0 (3.0–89.0)
Mean ± SD	39.1 ± 24.4
Recurrence/progression, no. (%)	9 (47.4)
Neurological outcome, no. (%)	
Stable	10 (52.6)
Worsened	9 (47.4)
Death during FU	8 (42.1)
PFS, mo	
Median (range)	32.0 (3.0–65.0)
Mean ± SD	31.8 ± 18.0
1-year PFS	89.2
3-year PFS	57.3
5-year PFS	20.5
OS (mo)	
Median (range)	35.0 (3.00–89.0)
Mean ± SD	39.1 ± 24.4
1-year OS	89.5
3-year OS	57.5
5-year OS	32.9

AR = aggressive resection, FU = follow-up, GTR = gross total resection, KPS = Karnofsky Performance Scale, mos = months, OS = overall survival, PFS = progression-free survival, PR = partial resection, STR = subtotal resection.

## 8. Preoperative radiological evaluation and diagnosis

Fifteen patients had both preoperative CT and MRI scans, 1 patient did not have the MRI enhancement scan, and the remaining 3 patients had MRI scans only. Radiologic findings are given in Table 3. Overall, CT and MRI scans were available for radiological evaluation for 16 and 18 patients, respectively. On CT scans, osteolytic changes were demonstrated in 16 cases (84.2%), and calcification was demonstrated in 3 cases (15.8%). The most common manifestations on MRI were isointense on T1-weighted images (8 cases [42.1%]) and T2-weighted images (7 cases [36.8%]). MRI had an absence of edema (16 cases [84.2%]) on T2-weighted images, with heterogeneous enhancement (14 cases [73.7%]) detected frequently. Overall, most lesions were derived from the skull base and mainly arose from the saddle area. Preoperative radiological diagnosis included meningioma (8 cases), followed by chordoma (3 cases), and pituitary tumor (3 cases).

**Table 3:** Tumor characteristics of 19 adenoid cystic carcinomas.

Variable	Value (%)
Location	
Skull base	17 (89.5)
Frontal lobe	1 (5.3)
Temporal lobe	1 (5.3)
Shape	
Flat	2 (10.5)
Irregular	14 (73.7)
Oval	3 (15.8)
Brainstem compression*	
Yes	3 (15.8)
No	16 (84.2)
MRI feature	
Hypo T1 and hyper T2	5 (26.3)
Mixed T1 and mixed T2	5 (26.3)
Iso T1 and iso T2	7 (36.8)
Iso T1 and hyper T2	1 (5.3)
Hyper T1 and hyper T2	1 (5.3)
Peritumor edema	3 (15.8)
Enhancement	
Heterogeneous	14 (73.7)
Homogeneous	4 (21.1)
NA	1 (5.3)
CT density	
Iso-density	4 (21.1)
Hypo-density	2 (10.5)
Hyper-density	4 (21.1)
Mixed-density	6 (31.6)
NA	3 (15.8)
CT feature	
Bone destruction	17 (84.2)
Calcification	3 (15.8)
Neither	1

NA = not available.

* Values were assessed using a combination of magnetic resonance imaging and intraoperative findings.

## 9. Surgical and pathologic findings

Twenty-six surgical procedures were performed among this cohort: once in 16 patients, 3 times in 2 patients, and 4 times in 1 patient. Various surgical approaches were used, as summarized in Table 4. Under a microscope, the lesions varied in color from grayish-red (12 cases) to grayish-white (3 cases), dark red (1 case), fuchsia (1 case), and grayish-yellow (1 case). The surgical plane was distinguishable in 11 lesions and was evident in only 8 lesions. The tumor consistency was soft and subjected to suction in 3 cases, tenacious in 12 cases, and hard in 4 cases. Intraoperative blood loss volumes ranged from 100 to 2000 mL, with a mean of 444.0 ± 445.0 mL. Intraoperatively, only 57.9% of tumors (11/19) were purely epidural without dura matter breakthrough, whereas the remaining 8 tumors were subdural and epidural (42.1%; 8/19). Based on postoperative MRI scans, GTR, subtotal resection, and PR were performed in 8 (42.1%), 6 (31.6%) and 5 (26.3%) patients, respectively. Postoperatively, 15.8% of patients (3/19) experienced various surgical complications that were described in Table 3. At discharge, neurologic status was stable in 52.6% of patients (10/19) and deteriorated in 47.4% of patients (9/19).

**Table 4:** Surgical results of adenoid cystic carcinoma.

Variable	Value (%)
Surgical approach	
Endoscopic trans-nasal approach	10 (62.6)
Frontotemporal cutting the zygomatic arch approach	4 (21.1)
Frontal orbital approach	2 (10.5)
Frontotemporal approach	1 (5.3)
Double frontal approach	1 (5.3)
Distal lateral approach	1 (5.3)
Extent of surgery	
Gross total resection	8 (42.1)
Subtotal resection	6 (31.6)
Partial resection	5 (26.3)
Relationship with dura mater	
Epidural	11 (57.9)
Subdural and epidural	8 (42.1)
Border of tumor	
Well defined	8 (42.1)
Poorly defined	11 (57.9)
Blood loss (mL)	
Median (range)	300 (100–2000)
Mean ± SD	444 ± 445
Tumor color	
Grayish-red	12 (63.2)
Grayish-white	3 (15.8)
Grayish-yellow	1 (5.3)
Fuchsia	2 (10.5)
Dark red	1 (5.3)
Blood supply	
Abundant	13 (68.4)
Moderate	6 (31.6)
Tumor consistency	
Tenacious	12 (63.2)
Hard	4 (21.1)
Soft/crisp	3 (15.8)
Surgical duration (minutes)	
Median (range)	265 (20.0–595)
Mean ± SD	277 ± 150
Surgical morbidities	3 (15.8)
Cerebrospinal fluid leak	2 (10.5)
Intracranial infection	1 (5.3)
CN VI	1 (5.3)
Coma	1 (5.3)
Outcome at discharge	
Stable	10 (52.6)
Worsened	9 (47.4)

CN = cranial nerve.

Immunohistochemical analysis was available in 6 patients. Cytokeratin 5/6 positivity was identified in 83.3% of patients (5/6); EMA, calponin, and S-100 positivity were found in 66.7% (4/6) of tumors; SMA, cytokeratin 8/18, cytokeratin, and Ki-67 positivity were observed in 50.0% of tumors (3/6); CEA and vimentin positivity were identified in 33.3% of tumors (2/6), and GFAP positivity was identified in 16.7% of tumors (1/6).

## 10. Follow-up and factors related to PFS or OS

No patients were lost to follow-up. Twelve (63.2%) patients received RT postoperatively: conventional fractionated RT in 9 patients, proton RT in 1 patient, and gamma knife RT in 2 patients. Unfortunately, the precise RT dose was unavailable because those patients accepted RT in other centers. Four (21.1%) patients were prescribed adjuvant chemotherapy after surgery. After a mean follow-up of 39.1 ± 24.4 months (range 3.0–89.0 months), the recurrence occurred in 1 patient (5.3%) with a PFS time of 41.0 months. Three patients (15.8%) had metastasis to intracranial, liver, and chest with a mean PFS time of 37.0 ± 4.0 months.

The median PFS was 31.8 months (range, 3.00–65.0 months). The 1-, 3-, and 5-year PFS rates were 89.2%, 57.3%, and 20.5%, respectively (Table 2 and Fig. 5H). Statistical testing showed that there were no significant difference in mean PFS between the GTR subgroup and Non-GTR subgroup (28.0 vs 34.6 months; *P* = .442), between patients who received and those who did not receive CMT (41.0 vs 29.4 months; *P* = .205), between patients who underwent RT postoperatively and their counterparts who did not (32.8 vs 30.3 months; *P* = .815), between patients who underwent surgery and surgery plus RT (30.3 vs 28.6 months; *P* = .8772), between patients who underwent surgery and surgery plus RT and CMT (30.3 vs 41.00 months; *P* = .376), and between patients who underwent surgery plus RT and surgery plus RT and CMT (28.6 vs 41.0 months; *P* = .179).

Eight patients (42.1%) had died. OS rates at 1-, 3-, and 5-years were 88.9%, 61.5%, and 35.2%, respectively (Table 2 and Fig. 5H). The median OS for those patients was 35.0 months (range, 3.0–89.0 months). Besides, no significant difference in mean OS was observed between the GTR subgroup and non-GTR subgroup (33.8 vs 42.9 months; *P* = .424), between patients who underwent CMT postoperatively and their counterparts who did not (50.8 vs 35.9 months; *P* = .327), between patients who underwent RT postoperatively and their counterparts who did not (41.2 vs 35.4 months; *P* = .669), between patients who underwent surgery and surgery plus RT (35.4 vs 36.4 months; *P* = .945), between patients who underwent surgery and surgery plus RT and CMT (35.4 vs 50.8 months; *P* = .384), and between patients who underwent surgery plus RT and surgery plus RT and CMT (36.4 vs 50.8 months; *P* = .349).

## 11. Discussion

ACC accounts for approximately 1% of all malignancies of the head and neck region.^[4]^ ACC is most frequently found in the oral cavity, primarily in the minor salivary glands, and occurs less frequently in the cranial base of the brain.^[5]^ They have diverse histopathological features, the 3 common types of growth patterns are cribriform (glandular), tubular, and solid. Among these 3 types, the solid pattern has the worst prognosis.^[6]^ ACC was characterized by insidious local growth, high recurrence rates, and distant metastasis. From an etiological perspective, intracranial ACCs have either a primary or secondary origin. Initially, the majority of primary ACCs are found at the skull base. In contrast, secondary intracranial ACCs are mainly caused by metastatic tumor emboli from other places of the body. In the study, 19 patients were primary while one patient had a recurrence, and 3 patients had the metastasis at the follow-up. Close follow-up was necessary for detecting intracranial ACCs recurrence or metastasis. The newly developed clinical symptoms should be considered essential indicators.

Intracranial spread can occur due to perineural growth, direct extension, and hematogenous spread. The first 2 modes were commonly reported, and some authors described a 50% incidence of perineural invasion in ACCs.^[1]^ Hematogenous spread is very unusual, with only 14 cases reported in available English literature.^[1]^ In this study, we found that 6 patients had perineural growth or direct extension, with 2 patients had hematogenous spread.

The nonspecific presenting symptoms provided a few criteria to make an accurate diagnosis. Even with the assistance of radiologic examinations, none of these lesions were preoperatively diagnosed as ACC. Preoperative diagnosis of meningioma, chordoma, and pituitary tumor in the series accounted for 42.1%, 15.8%, and 15.8% of lesions, respectively. The relatively low rate of correct preoperative diagnosis was also attributed to a broad spectrum of MRI features. Fortunately, ACC could be diagnosed if unique, biphasic histology composed of areas of classic cribriform growth pattern, like nests of tumor cells with discrete, rounded, “punched‑out” gland‑like spaces filled with either eosinophilic or basophilic material, and the round or oval tumor cells that were similar to basal cells. Based on our study, preoperative radiological data are less reliable and insufficient for accurate diagnosis; additionally, the pathological examination should be used to determine the diagnosis.

### 11.1. Clinical features and radiological features

Clinically, intracranial ACCs can present with features of raised intracranial pressure, focal neurological deficits, or a combination of both. Furthermore, the intracranial ACCs mostly originate from the skull base (16 cases [84.2%]); as a consequence, CN palsy is the chief complaint. Patients with lesions involving the jugular foramen and neck, patients often present difficulty swallowing or difficulty drinking. When lesions invaded the optic canal, CNII defects often developed (7 cases [36.8%]). When temporal bone lesions invade posteriorly, patients often present with CNV palsy (6 cases [31.6%]). Peak incidence occurs predominantly among women, between the fifth and sixth decades of life.^[7]^ In contrast, patients in our series were predominantly male, with a male/female ratio of 10:9, which was similar to the ratio in the literature,^[8]^ and the mean and median ages were 47.3 ± 12.2 and 51.0 years, respectively. The survival rates at 5 and 10 years are approximately 50% and 20%, respectively.^[6]^ During the first 5 years of follow-up, local treatments suggest a high success rate, with 50% to 75% remaining disease-free.^[4]^ In contrast, the 1-, 3-, and 5-year rates of PFS and overall survival were 89.2%, 57.3%, and 20.5% and 89.5%, 57.5%, and 32.9%, respectively.

In primary ACCs, p53 has shown inconsistent and unreliable expression, but in recurrent tumors, its overexpression has been seen quite often. Another marker expressed in ACC with or without perineural spread is the brain‑derived neurotrophic factor.^[9]^ E-cadherin has been shown to have reduced expression in progressive forms of ACC.^[1]^ In the cohort, the main immunohistochemistry result was positive for Cytokeratin5/6, EMA, calponin, S-100, SMA, Cytokeratin8/18, Cytokeratin, Ki-67, CEA, Vimentin, and GFAP.

Da Cruz Perez et al^[5]^ noted that pathology examination is a decisive method for diagnosing ACC. CT scans can reveal bone destruction and the clearness of tumor margins. ACC accounts range from hypodense to hyperdense on CT scans. Bone window imaging reveals the degree of bony destruction (17 cases [89.5%]), calcification (3 cases [15.8%]), and the expansile pattern of the mass and bony growth is not found with ACCs. Generally, these tumors are usually hypointense or strongly contrast-enhancing on T1 weight image and hyperintense on T2 weight image.^[10–12]^ However, the imaging features of ACCs in our case were very distinctive (Table 3). The low signal intensity, iso signal intensity, and high signal intensity and mixed-signal intensity on T1-weighted images were 26.3%, 42.1%, 5.3%, and 26.3%, respectively. The iso signal intensity, high signal intensity, and mixed-signal intensity on T2-weighted images were 36.8%, 36.8%, and 26.3%, respectively. MRI had an absence of edema (84.2%) on T2-weighted images, with heterogeneous enhancement (73.7%) detected frequently.

## 12. Proposed treatment paradigm

Data that are focused on the treatment of intracranial ACC are limited. The currently used treatments are safe maximal cytoreduction followed by adjuvant RT, which is based on its aggressive behavior on histopathology and high recurrence rate.^[13]^ The majority of intracranial ACCs in the pooled cohort originated in the skull base. Because of their mucinous content, incomplete encapsulation, and proximity to essential structures, only 8 cases (42.1%) from our institute had GTR. Complete removal of lesions from the cranial base bone is difficult, especially when the lesions have widely and deeply infiltrated the skull base. Even if complete removal were feasible, the morbidity and mortality rates would be too high. Surgery aims to obtain disease-free surgical margins and, at the same time, to maintain the normal function of the affected structures, when this is possible. The goal of surgery for the intracranial ACCs in our series was to debulk the tumor and to confirm the diagnosis pathologically to guide subsequent treatment strategy. Furthermore, following gross-total removal, reconstructing the cranial base can also be a challenge.

For the remaining 15.8% of ACCs in our cohort and not in the skull base, especially when the tumor was not near eloquent areas, radical surgical removal should be tried if possible. The goal of surgery in such circumstances theoretically is to achieve a clear resection margin. It was not surprising that the ETR did not affect PFS or OS in this cohort, which could be explained by the failure to determine whether clear resection margins were achieved even in the GTR group. Surgery alone, even when it is radical, can rarely avert local recurrences because of the frequent presence of infiltration into the surrounding soft tissues (perivascular and perineural invasion) and bone.^[4,14]^

This tumor is radiosensitive, but not radio-curable,^[14,15]^ and advanced adjuvant RT, neutron beam or proton therapy, could yield good outcomes for patients with intracranial ACC.^[6,16,17]^ Radiotherapy in ACC can increase the number of patients with locoregional control when it is given postoperatively. Notably, a longer mean PFS (32.8 vs 30.3 months; *P* = .815) and OS (41.2 vs 35.4 months; *P* = .669) were observed in patients with RT than in those without RT.

Chemotherapy in ACCs is usually confined to advanced disease, that is, nonresectable, recurrent, or metastatic disease. Unlike for RT, the effect of CMT on the prognosis for intracranial ACCs is still controversial. In 2019, Tchekmedyian et al thought the lenvatinib elicited promising efficacy in ACCs.^[18]^ In an extensive review conducted by Dodd and Slevin in 2006, it was observed that although the objective response rate to chemotherapy is low, the symptomatic responses are generally higher.^[19]^ Moreover, from the review of the efficacy of different chemotherapy regimens, we can conclude that response rates to chemotherapy are low, and response duration is generally short-lived.^[4]^ However, In this pooled analysis, we found that a longer mean PFS of patients treated with CMT than that of patients treated without CMT (41.0 vs 29.4 months; *P* = .205), and a trend of better OS were also found in the CMT subgroup (50.8 vs 35.9 months; *P* = .327). The administration of chemotherapy as one part of multimodality treatment for intracranial MCS is worth trying.

Although few convincing studies have been performed, a longer mean PFS (30.3 vs 41.00 months; *P* = .376; 28.6 vs 41.0 months; *P* = .179) and OS (35.4 vs 50.8 months; *P* = .384; 36.4 vs 50.8 months; *P* = .349) were observed in patients with surgery than in those with surgery plus RT and CMT and surgery plus RT than in those with surgery plus RT and CMT in our series. Combined with other evidence, a combination of RT and chemotherapy is considered applicable.

### 12.1. Study limitations

As with all studies, there are some weaknesses and limitations in the current study. First, it is a retrospective review of case reports; thus, the rate of missing detailed data for survival analysis is relatively high. The literature has a mean follow-up time of 39.1 ± 24.4 months, which cannot reflect a reliable prognostic result. Second, given the rarity of intracranial ACCs, there was a lack of randomized data, and potential bias may exist in the statistical analysis data. Third, the detail of RT and CMT used in this cohort was insufficient. Besides, we could not identify a significant predictor for OS or PFS because of the relatively small cohort.

The subjective consistency measurement reflected a single-institute experience and was a limitation of the study; therefore, we defined it as a semi-quantitative consistency measurement according to intraoperative findings and the hardest part of the lesion. Given the intralesional heterogeneous consistency and the retrospective nature of the study, an objective quantitative consistency measurement was unavailable. Therefore, a prospective multicenter study with a large series of intracranial ACCs is recommended.

## 13. Conclusions

The clinical characteristics of intracranial ACCs are variable. The current management of intracranial ACCs did not yield satisfactory outcomes. Intracranial ACCs are rare neoplasms with a slightly higher occurrence in males. GTR, if tolerable, is advocated as the optimal treatment for intracranial ACCs. Nevertheless, conservative excision may be preferred to avoid damage to vital structures. Intracranial ACCs tend to recur or metastasis within a few years of the initial surgery; therefore, postoperative follow-up is essential. Radiotherapy and chemotherapy may be an alternative treatment. Further study with large cohorts is needed to confirm the efficacy of chemotherapy and more effective RT, including SRS and proton therapy.

## Acknowledgments

We thank Prof Jiang Du (Department of Pathology, Beijing Neurosurgical Institute, Beijing Tiantan Hospital) for the support with the histopathologic analysis.

## Author contributions

**Conceptualization:** Xu-Lei Huo, Da Li.

**Data curation:** Xu-Lei Huo, Xi-Chao Yin.

**Formal analysis:** Xu-Lei Huo, Ke Wang.

**Funding acquisition:** Ke Wang.

**Investigation:** Zhen Wu.

**Methodology:** Xu-Lei Huo, Zhen Wu, Ke Wang.

**Project administration:** Zhen Wu, Ke Wang.

**Resources:** Xu-Lei Huo, Zhen Wu.

**Software:** Xu-Lei Huo, Ke Wang.

**Validation:** Xu-Lei Huo, Ke Wang.

**Visualization:** Ke Wang.

**Writing – original draft:** Xu-Lei Huo, Ke Wang.

**Writing – review & editing:** Xu-Lei Huo, Ke Wang.
